# ASO Author Reflections: Humidification of Carbon Dioxide for Insufflation Offers Promise in Reducing Inflammation and Peritoneal Trauma During and Post Colorectal Cancer Surgery

**DOI:** 10.1245/s10434-022-13014-w

**Published:** 2023-01-02

**Authors:** Robert Ramsay, Shienny Sampurno, Michael P. Flood, Andrew C. Lynch

**Affiliations:** 1 Sir Peter MacCallum Department of Oncology, University of Melbourne, Melbourne, VIC Australia; 2 Department of Surgical Oncology, Peter MacCallum Cancer Centre, Melbourne, VIC Australia; 3 Epworth Healthcare, Richmond, VIC Australia

## Past

The use of insufflation to generate a pneumoperitoneum and facilitate laparoscopic and robotic surgery is routine practice. Inflation of the abdomen is typically achieved using medical grade carbon dioxide (CO_2_), delivered directly from a cylinder as a dry and cold (D-C-CO_2_) gas. Informed by the benefits of humidifying other gases in the context of anesthetic and perinatal care^1,2^ and preclinical studies, investigators and manufacturers have explored the effects of CO_2_ on a range of clinical parameters. The evidence from animal studies is convincing and reproducible. It shows that humidified-warm CO_2_ (HW-CO_2_), on balance, leads to less negative impact on core body temperature, post-surgical adhesions, and peritoneal damage.^3,4^ By contrast, clinical studies including randomized clinical trials (RCTs) examining pain control and length of hospital stay (LOS) are not as compelling.^5^ Underpinning this dichotomy are the types of issues that might be addressed, including biological sampling, surgical protocols, and patient heterogeneity. For instance, it is challenging to measure pain in patients but even more so in rodent models. On the other hand, it is easy to sample rodent tissues over times ranging from hours to days, while this is limited to the operative period in patients.

## Present

In an attempt to align animal and patient outcomes, we designed and initiated a clinical RCT with a particular emphasis on peritoneal damage, core body temperature, and systemic inflammation, particularly because peritoneal tissue samples can be harvested with minimal patient impact, and, when subjected to scanning electron microscopy, can be evaluated in an unbiased, blinded fashion. Similarly, core body temperature and inflammatory markers such as C-reactive protein (CRP) can also be recorded before, during, and after surgery by investigator-blinded data collection. The patient cohorts were confined to those having surgery for localized colorectal cancer, and 66 minimally invasive (laparoscopic and robotic) cases were randomized to dry-cold or humidified-warm CO_2_, respectively. Additionally, our original intent was to explore cases undergoing laparotomy. This was based on animal studies showing that flooding the open abdomen with HW-CO_2_ was protective compared with passive or active airflow in the animal model.^4,6^ However, only a few open patients (19) were recruited for a range of reasons, but they served as an informative sample. Overall, we found that peritoneal damage was significantly reduced in the HW-CO_2_ cohort compared with the DC-CO_2_ cohort. Core body temperatures returned to normothermia faster in HW-CO_2_ patients, and CRP levels were significantly lower at postoperative days 1–4 in this group.^7^

## Future

As with most studies, new questions arise, one of which is potentially significant, i.e. the presence of peritoneal damage on initial biopsy in 20–30% of cases. This was a genuine surprise, particularly because this was not found in mice or pigs. This observation may reflect the nature of preclinical studies where young, healthy animals are used, and by contrast, our surgical cases are not as young and often have comorbidities. However, variation in sampling technique or timing are not excluded. From a clinical perspective, the matter of enduring higher inflammatory markers in non-sepsis patients warrants deeper investigation, particularly in relation to the risk of developing peritoneal metastases, as suggested by animal models.^3^ Extended follow-up of this patient group to test the hypothesis that HW-CO_2_ can influence the development of peritoneal metastases would be informative. The potential reduction in LOS associated with the use of HW-CO_2_ is noted, and verification with a larger number of cases grouped into colon or rectal cancer surgery focusing on postoperative pain and LOS as primary outcomes would be useful. Finally, the findings at open surgery are promising and warrant further investigation.
